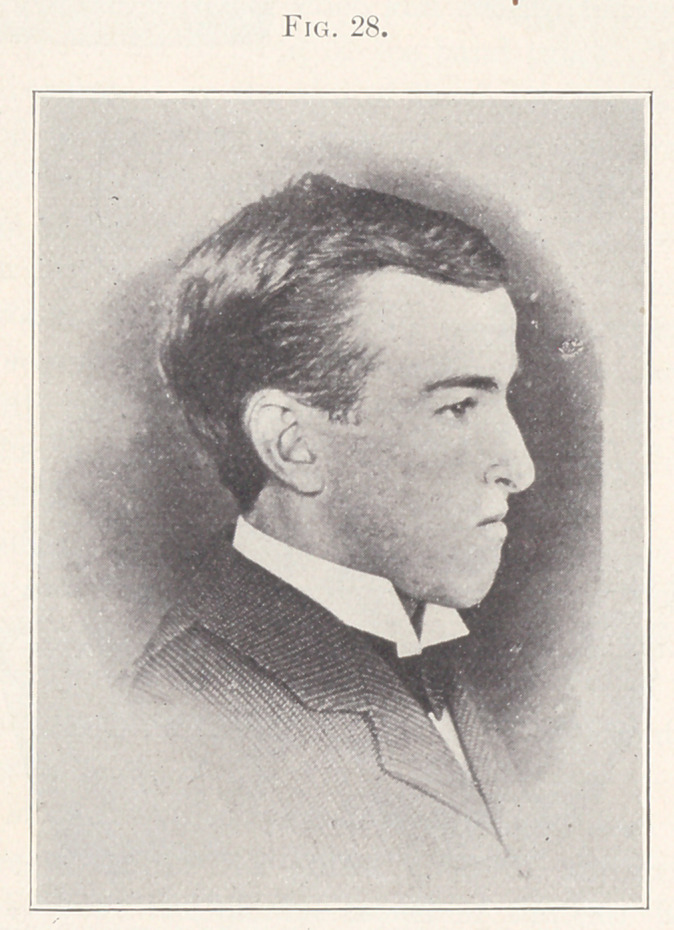# A Contribution to Operative Orthodontia

**Published:** 1902-08

**Authors:** Eugene H. Smith


### A CONTRIBUTION TO OPERATIVE ORTHODONTIA.[1]

BY EUGENE H. SMITH, D.M.D.[2]

Mr. President and Gentlemen of the Academy,—Of the four cases which I shall present to you this evening, the first two are from my private practice and the last two are from my orthodontia clinics in the Harvard Dental School, and are, I think, interesting inasmuch as they show the good results obtained by the use of intermaxillary elastics in connection with expansion wires and reciprocal anchorage, and also as a study of that debatable question,—namely, "The Jumping of the Bite."

The use of elastic rubbers with occlusal and reciprocal anchorage has for a long time been used in the correction of various forms of irregularities of the teeth, but the application of elastic rubbers with reciprocal anchorage, in the manner I have used them in three of the cases which I am about to show you, was, so far as I know, first made by Dr. H. A. Baker in the case of his son, and reported in "Angle," page 254.

Case I.—From my private practice. Patient, young lady, aged sixteen years. The nature of the irregularity is shown in

---

[1] Read before the American Academy of Dental Science, Boston, February 5, 1902.

[2] Professor of Orthodontia in the Harvard Dental School.

Fig. 1. In this case it seemed wise to widen both arches and carry forward the anterior teeth. For this purpose the usual expansion wires were used, and the result is shown in Fig. 2, which shows the two models open and in comparison, and in Fig. 3, showing the closed model and the present relation of the teeth, which condition, while presenting a pleasing appearance, is lacking in a normal occlusion.

As the necessary force in this case had to be outward in both the upper and lower teeth, the use of intermaxillary rubbers were contraindicated, but they could now be applied in an attempt to jump the bite forward, and the result would clearly indicate whether the change of occlusion would be obtained by any change in the glenoid cavity, or from a slight tipping of the teeth. Unfortunately for the further study of this case, the patient is away for the winter, and further regulating cannot, for the present, be done.

Fig. 3 also shows the present relation of the teeth.

Case II.—From my private practice. Girl, aged thirteen years. The nature of this irregularity is shown in Fig. 4. This irregularity required for its correction the retraction of the superior anterior teeth, widening of the arches, and the jumping of the bite forward. The appliance consisted of expansion wires on the teeth of both jaws and intermaxillary elastics. The result is shown in Fig. 5, also in Figs 6 and 7, showing the palatal aspect of Figs. 8 and 9 in comparison. Fig. 9 also shows the present relation of the teeth which lack the normal occlusion.

In the treatment of this case the teeth were purposely so fastened to the expansion wire as to prevent tipping, and the result shows that the lower jaw was brought forward very little, if any, and further, that the posterior anchorage teeth were not elongated nor tipped in the slightest degree.

Case III.—From the orthodontia clinics of the Harvard Dental School. Patient, boy, aged thirteen years, whose picture in profile you see on the screen. (Fig 10.) He was first seen by me in October, 1901, and assigned to a Senior student.

Fig. 11 shows a front view of the patient.

Fig. 12 shows a plaster model of the teeth and the condition before treatment, which is, as you see, a marked protrusion of the anterior teeth and an abnormal occlusion.

Fig. 13 shows the palatal aspect of the case. The first molars

Fig. 1.

Fig. 2.

Fig. 3.

Fig. 4.

Fig. 5.

Fig. 6.

Fig. 7.

Fig. 8.

Fig. 9.

Fig. 10.

Fig. 11.

Fig. 12.

Fig. 13.

Fig. 14.

Fig. 15.

Fig. 16.

Fig. 17.

Fig. 18.

Fig. 19.

Fig. 20.

Fig. 21.

on both jaws had been extracted and the spaces somewhat closed. The appliance used was expansion wire on the teeth of both jaws, banding the four second molars. On the upper the wire was threaded as far forward as the cuspids, and a sliding sleeve propelled by a small nut fixed in position. Resistance was strengthened by ligating the wire to all of the teeth save the second bicuspids, which were carried back by the force of the sliding sleeve to which they were ligated. The first bicuspids and cuspids were treated in the same way, and at the same time the arch was widened by the force of the band springing outward. On the lower, the anterior teeth were carried forward by various adjustments of the ligatures. Fig. 14 shows the lingual aspect of the case before treatment. At this stage intermaxillary elastics were fastened back of the lower tubes attached to the bands on the second molars and carried forward and ligated to the expansion upper wires at a point near the cuspids. In about four weeks this force retracted the protruding incisors and settled the teeth in good occlusion.

Fig. 15 shows the palatal aspect of the finished case.

Fig. 16 shows the lingual aspect of the finished case.

Fig. 17 shows the front view of the models before and after treatment.

Fig. 18 shows the profile view of the model after treatment.

Fig. 19 shows a picture of the patient, front view, after treatment.

Fig. 20 shows a profile view of the patient after treatment.

A study of this case leads me to believe that the change that has taken place in the relation of the teeth to each other is to be found in the movement of the teeth in their alveolar socket, rather than in the glenoid cavity of the jaw.

Case IV.—From the orthodontia clinics of the Harvard Dental School. Patient, young man, aged twenty-two years, whose picture you see upon the screen. (Fig. 21.) This patient was seen by me in October, 1901, and assigned to a Junior student.

Fig. 22 and the picture upon the screen show a case of pronounced prognathism in connection with a contracted and V-shaped upper arch, and is much like a case referred to in "Angle," page 179, in the following words:

"*Double Resection of the Maxilla.*—Several years ago the author became convinced that no operation depending upon tooth movement alone could establish proper relations of the teeth or materially improve the facial lines in certain cases of pronounced over-development of the inferior maxilla. It seemed to him that such cases might be successfully treated by the removal of a section of bone from each of the lateral halves, although the operation was not contemplated except as a remedy for the most aggravated conditions."

I do not claim that in this case we have made much, if any, change in the facial lines, so far as they relate to the over-development of the lower jaw, but we certainly have established proper relations of the teeth and improved the facial lines by the change produced in the upper arch.

On the upper jaw the first molars were missing and the second molars were in contact with the second bicuspids. On the lower jaw the first molars were missing, leaving a slight space between the second molars and bicuspids.

The appliances used to correct the deformity were expansion wires on the teeth of both jaws, using the second molars on either jaw for the bands and tubes into which entered the ends of the expansion wires, which were threaded and carried four nuts, which were placed on the wires in front of the band tubes. The anchorage was made reciprocal by the use of the intermaxillary elastics, and so attached to the wire as to exert a force outward on the upper teeth and backward on the lower teeth and jaw.

At one stage of the process it was found that the lower incisors were tipping in to the extent of carrying their roots out through the alveolar process, and to obviate this a double expansion wire was used, the second or lower wire bearing on the incisors near their necks, while the first or upper wire impinged near the cutting edges. The teeth were ligated to these wires with silk ligatures, which were changed every two or three days, and force applied here and there as needed. The first and second bicuspids and cuspids on the lower jaw were retracted by the use of the sliding sleeve and nut on the expansion wires. Figs. 23 and 24 show a condition of the occlusion during the process of treatment which was remedied by grinding occluding points and by elongating the anterior teeth.

At the end of May the glaring deformity had been corrected, and the pictures which we now throw upon the screen show the results. Fig. 25 shows the palatal aspect of the case before and after treatment.

Fig. 22.

Fig. 23.

Fig. 24.

Fig. 25.

Fig. 26.

Fig. 27.

Fig. 28.

Fig. 26 shows the front view of the models before and after treatment.

Fig. 27 shows the profile view of the model, completed case.

Fig. 28 shows the picture of the patient after treatment.

The study of this case also leads me to conclude that the change made was in the teeth and not in the temporo-maxillary articulation.

In the handling of the two cases from the clinics I had the valuable assistance of Dr. Lawrence W. Baker, assistant in orthodontia in the Harvard Dental School, who, besides seeing the cases with me on every Saturday throughout the course, also saw them on Mondays and Thursdays of each week. I am also indebted to Instructors Cross and Chute for their oversight in the construction of appliances and in the keeping of the records. In the cases from my private practice, I was assisted by my associate, Dr. Haley.

## Figures and Tables

**Fig. 1. f1:**
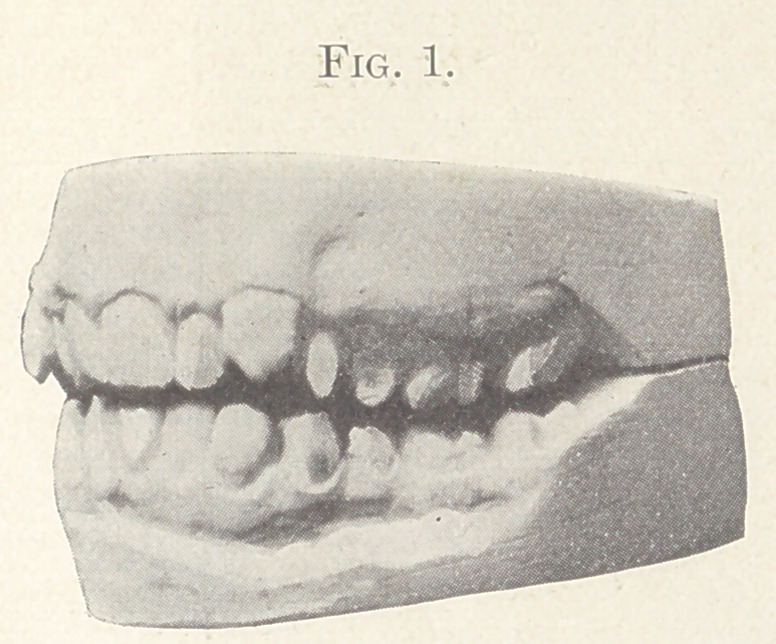


**Fig. 2. f2:**
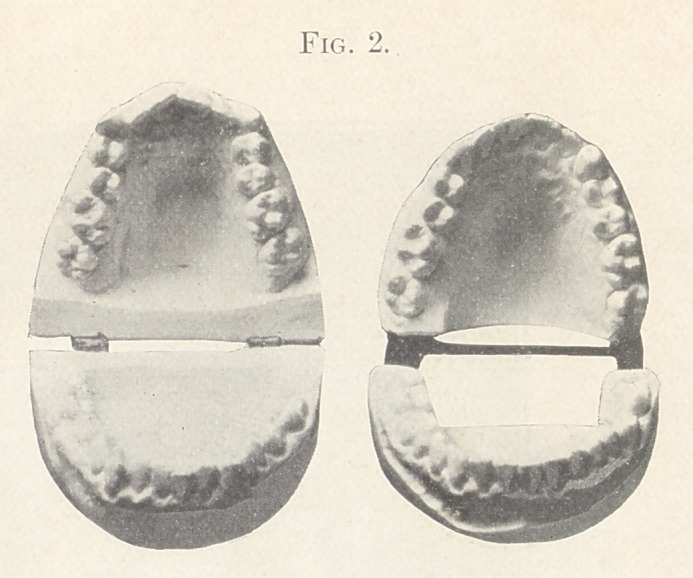


**Fig. 3. f3:**
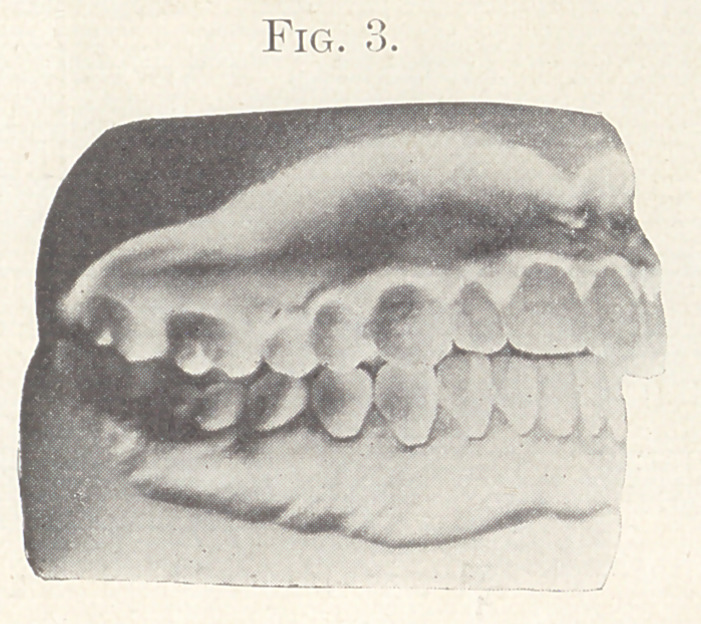


**Fig. 4. f4:**
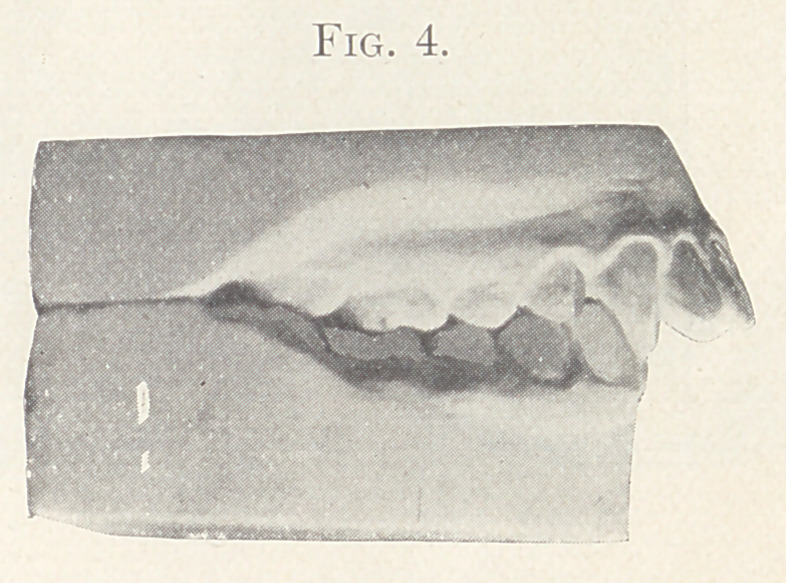


**Fig. 5. f5:**
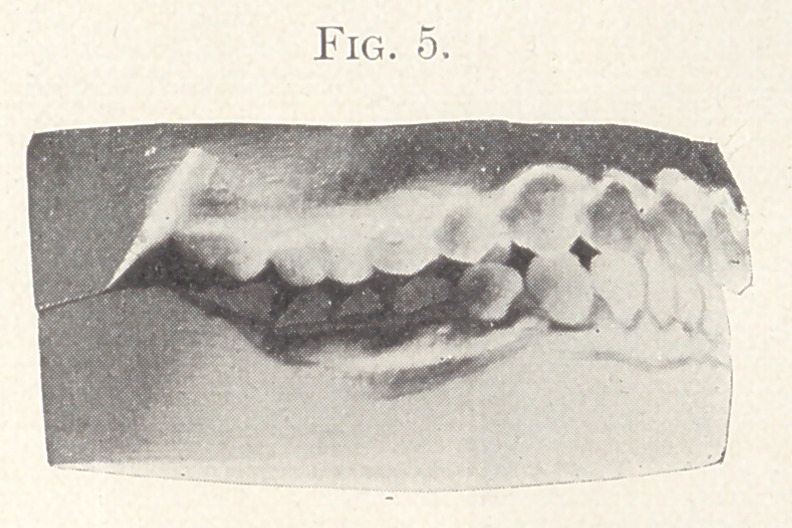


**Fig. 6. f6:**
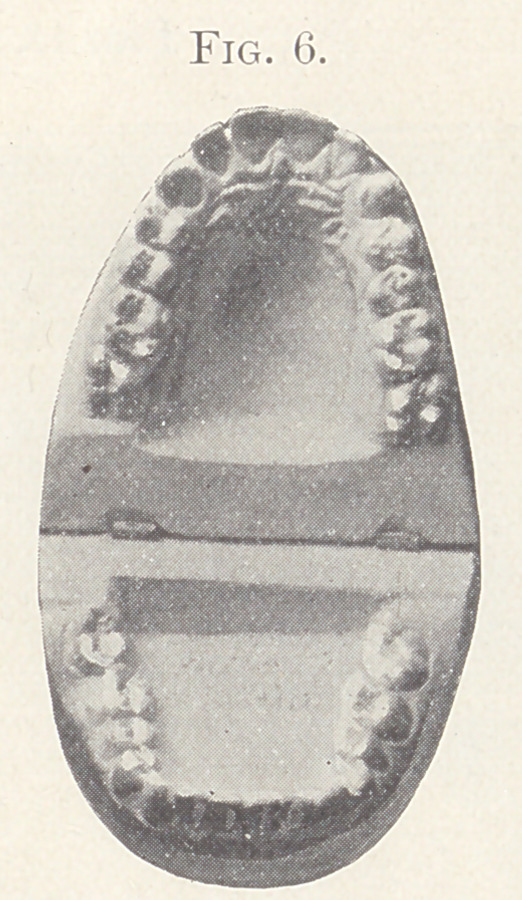


**Fig. 7. f7:**
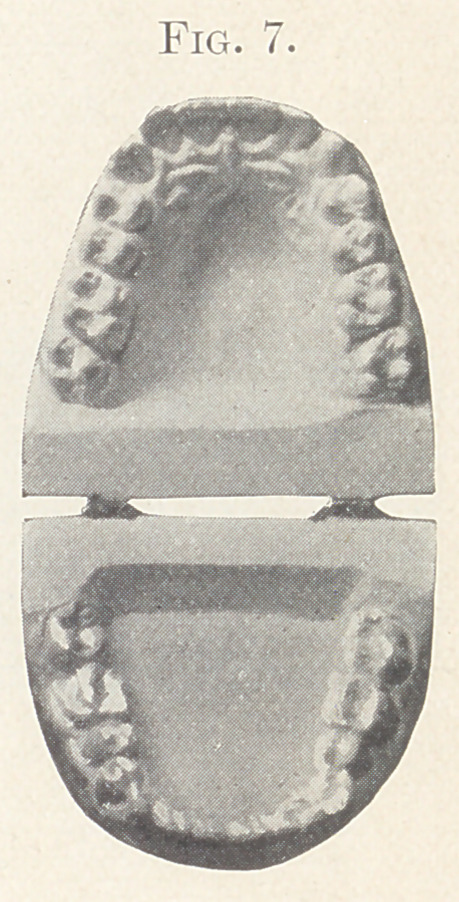


**Fig. 8. f8:**
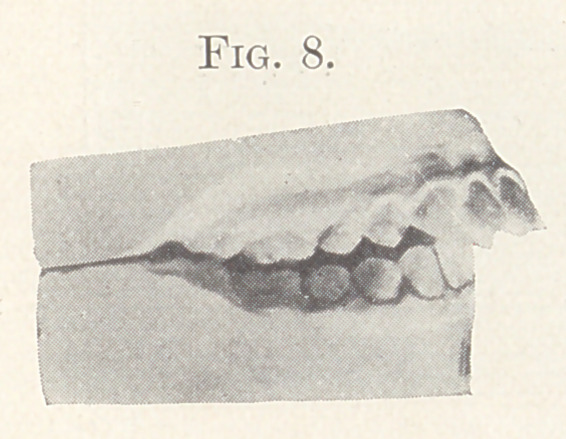


**Fig. 9. f9:**
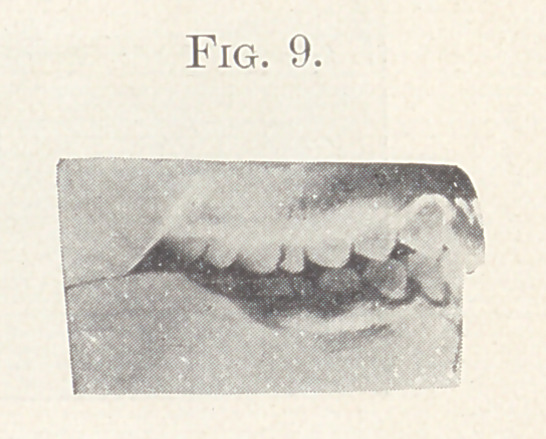


**Fig. 10. f10:**
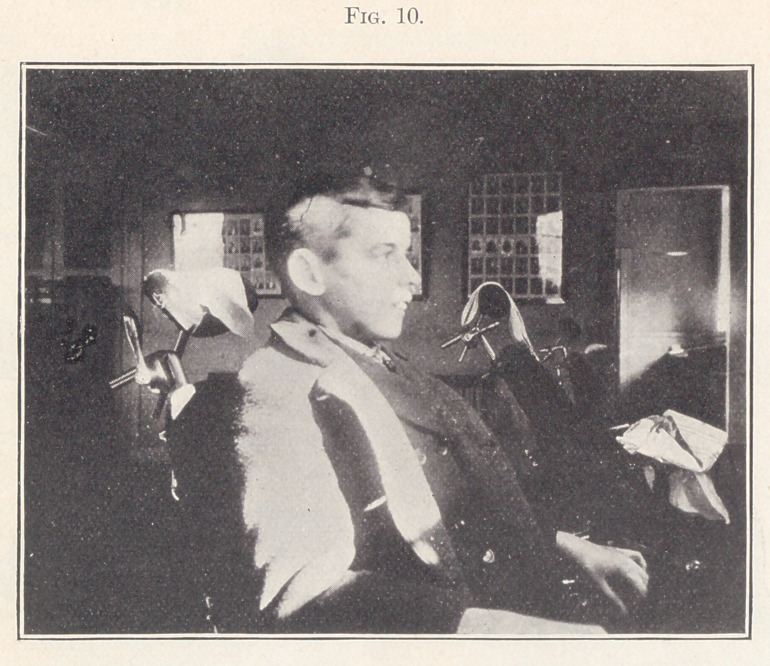


**Fig. 11. f11:**
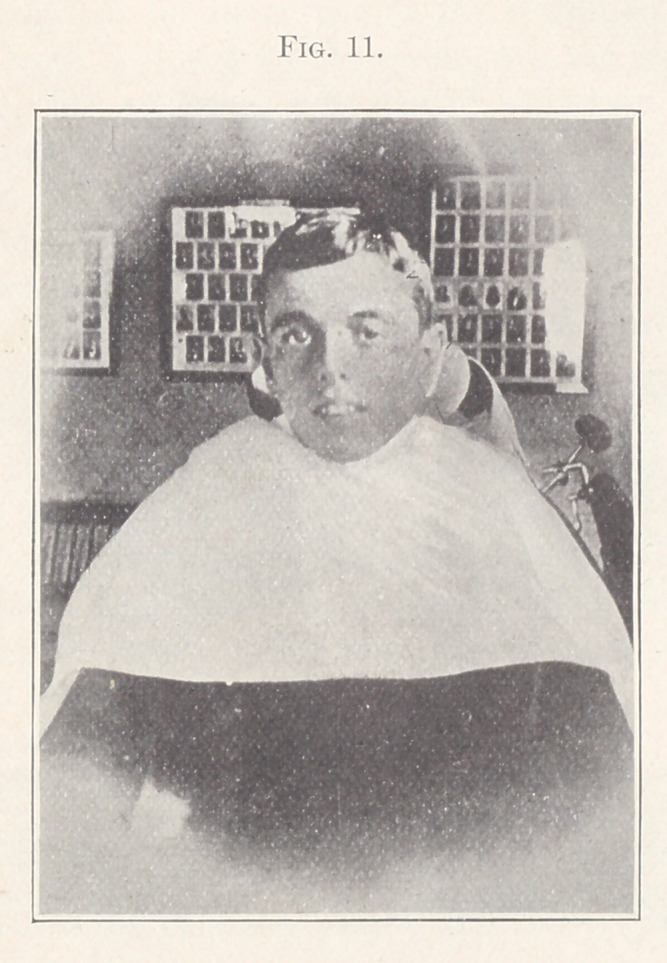


**Fig. 12. f12:**
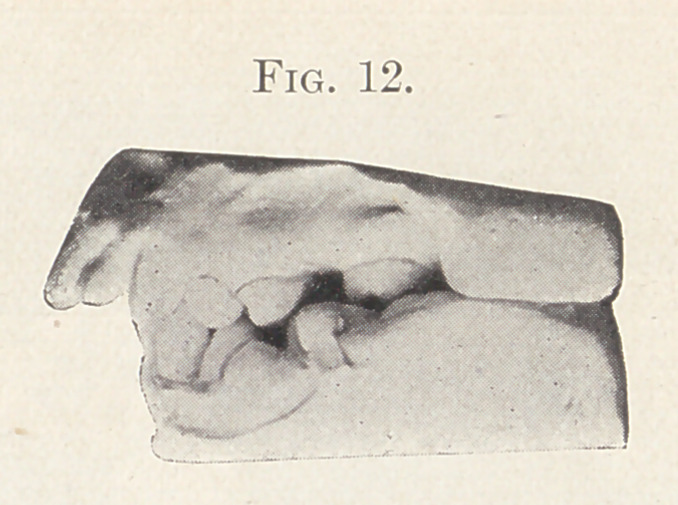


**Fig. 13. f13:**
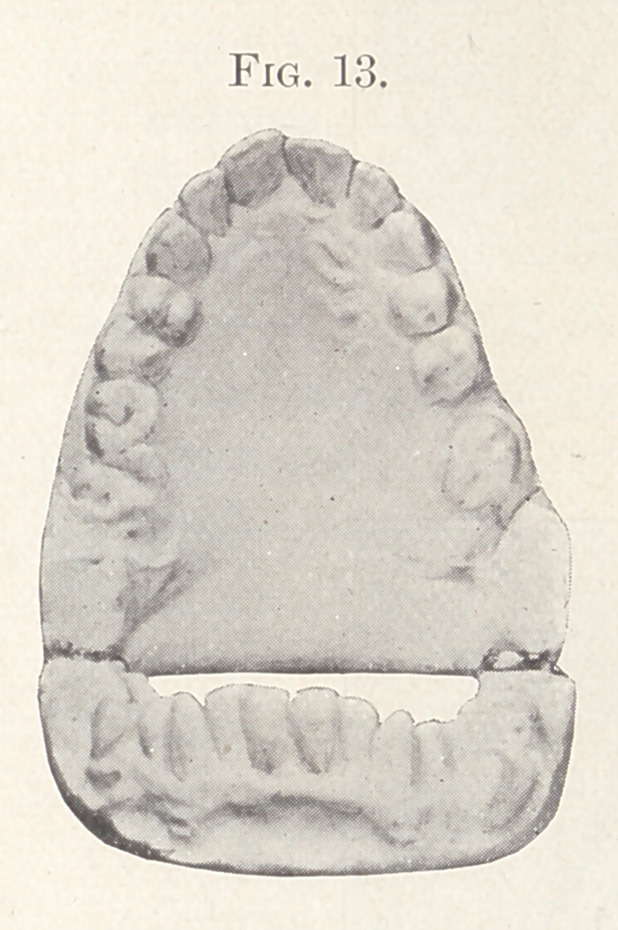


**Fig. 14. f14:**
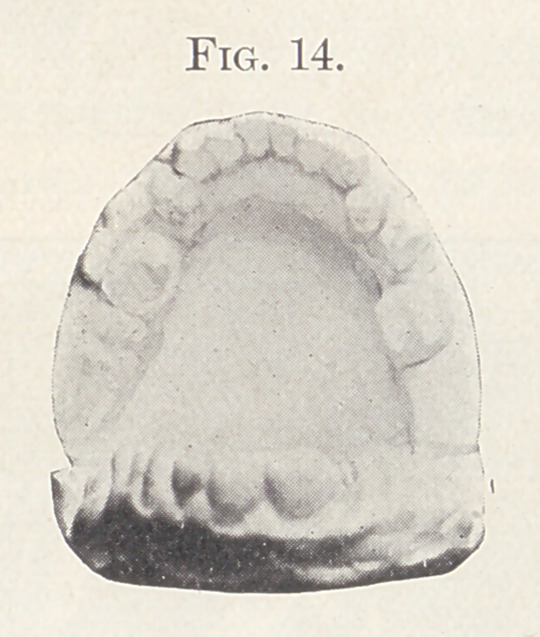


**Fig. 15. f15:**
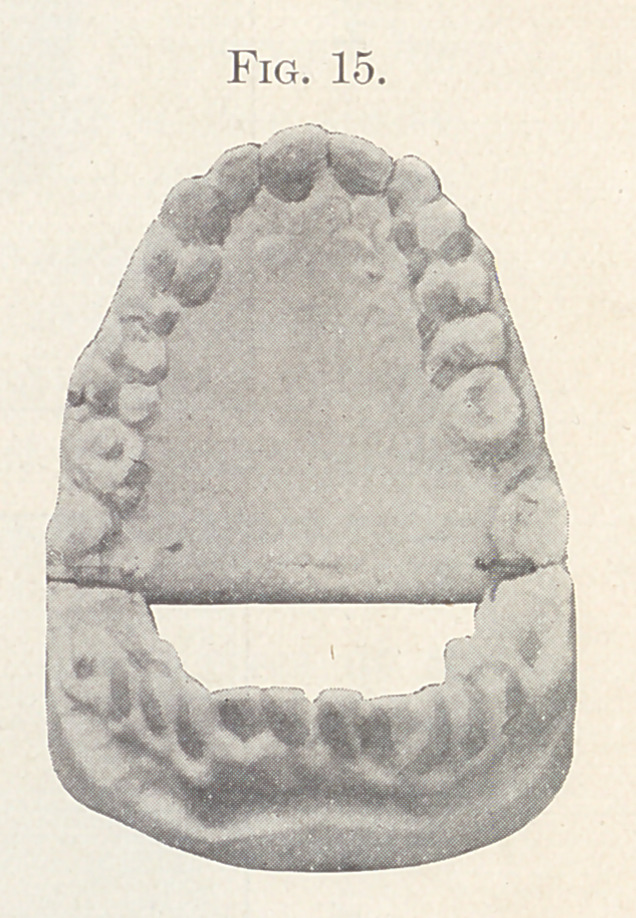


**Fig. 16. f16:**
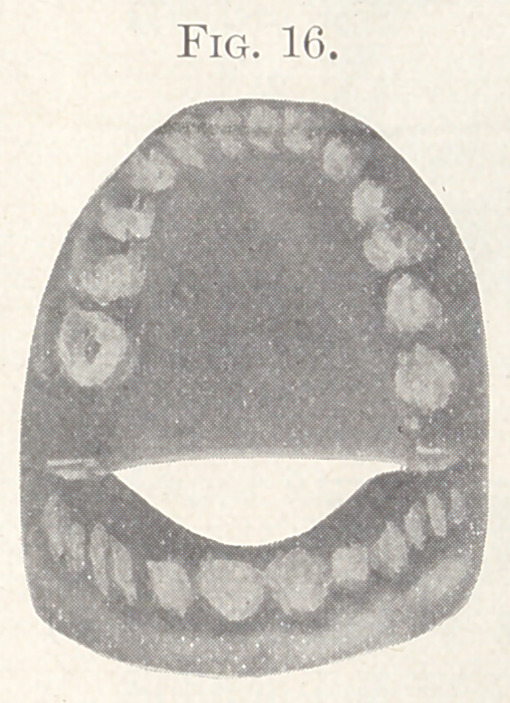


**Fig. 17. f17:**
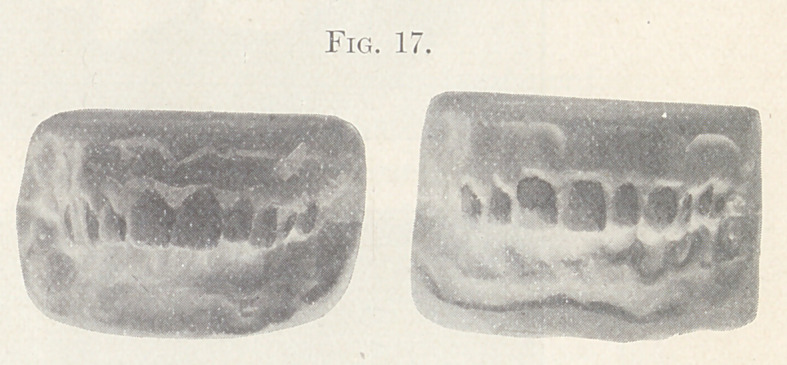


**Fig. 18. f18:**
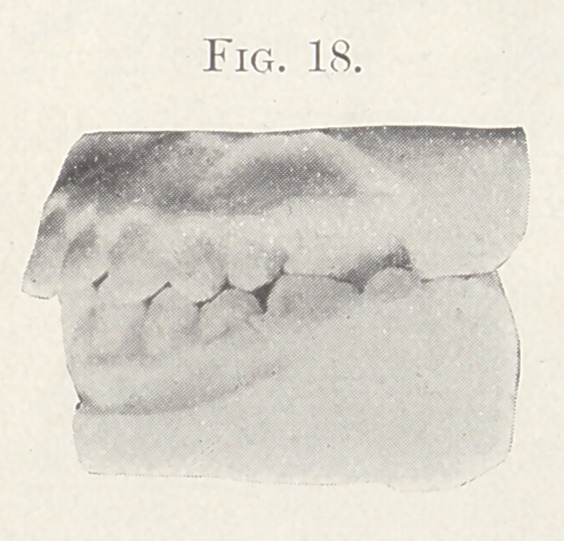


**Fig. 19. f19:**
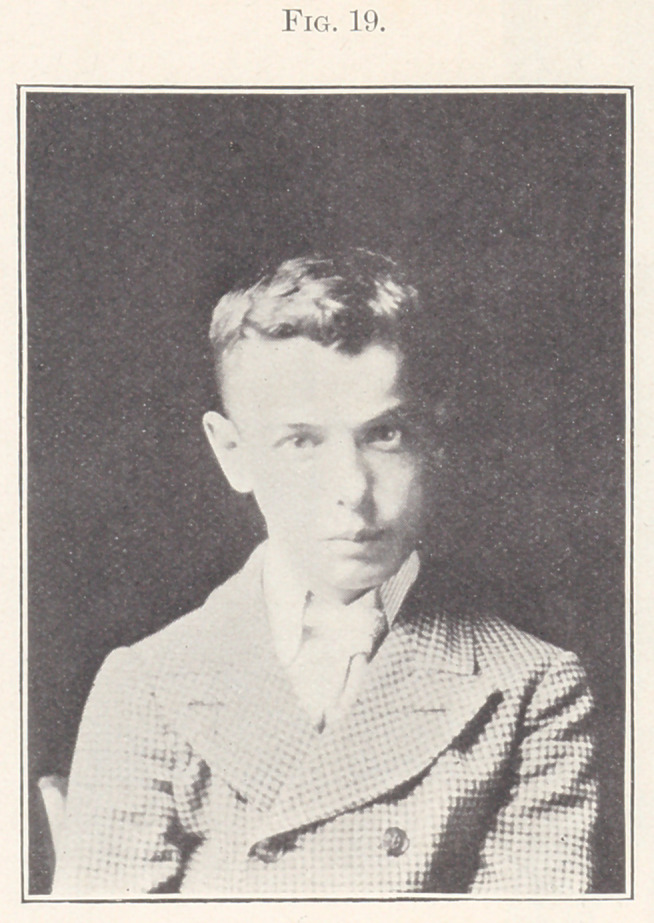


**Fig. 20. f20:**
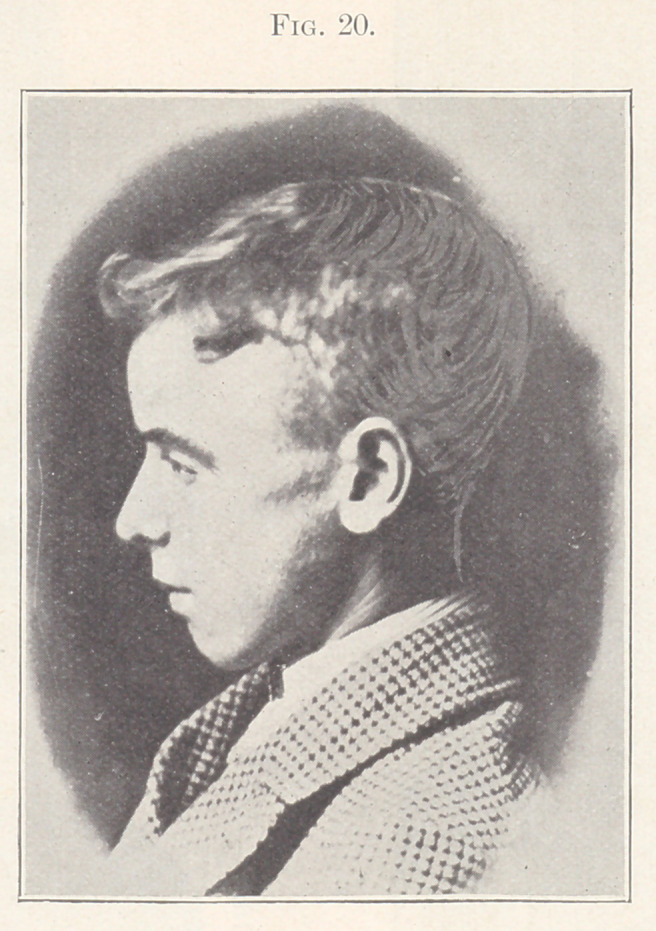


**Fig. 21. f21:**
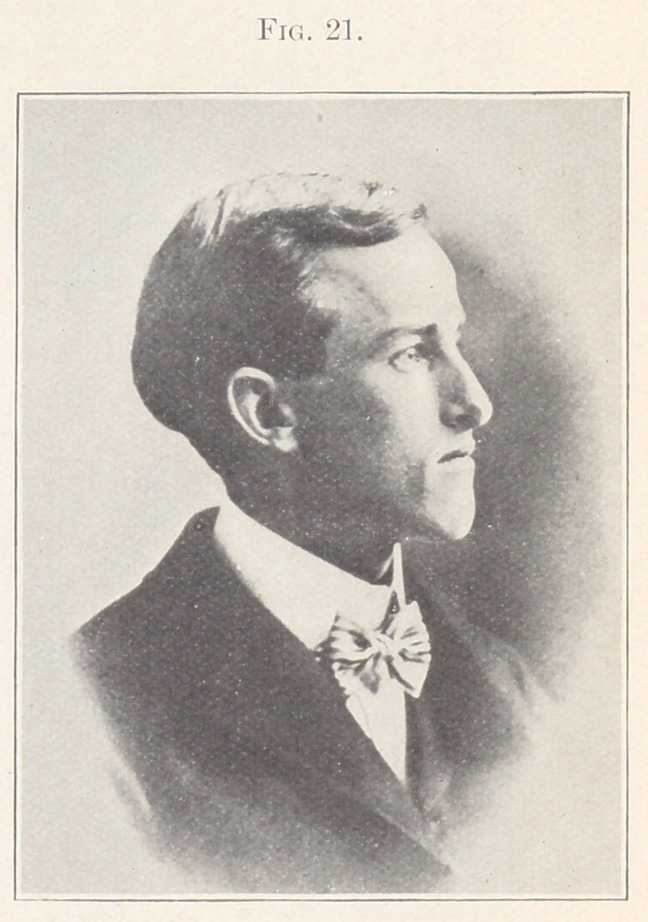


**Fig. 22. f22:**
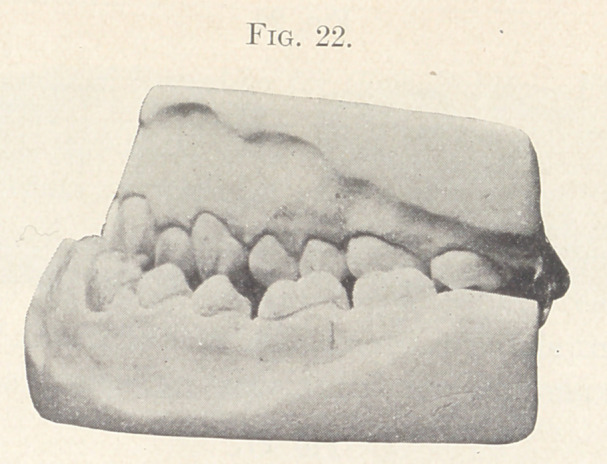


**Fig. 23. f23:**
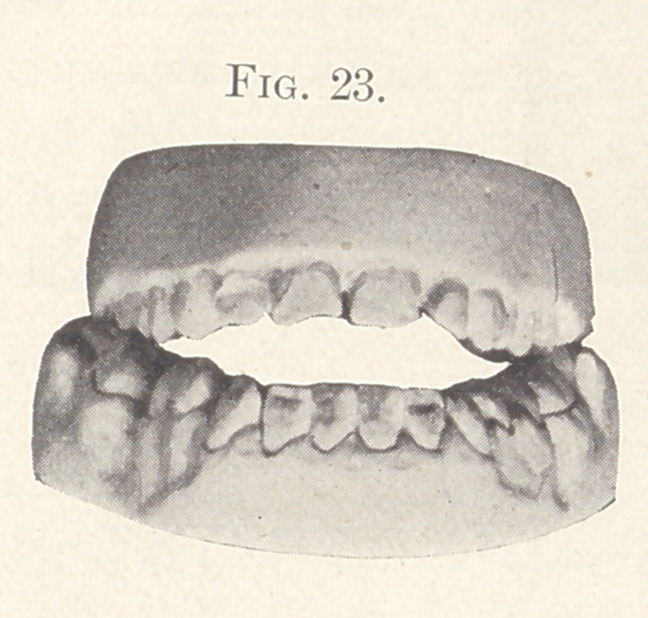


**Fig. 24. f24:**
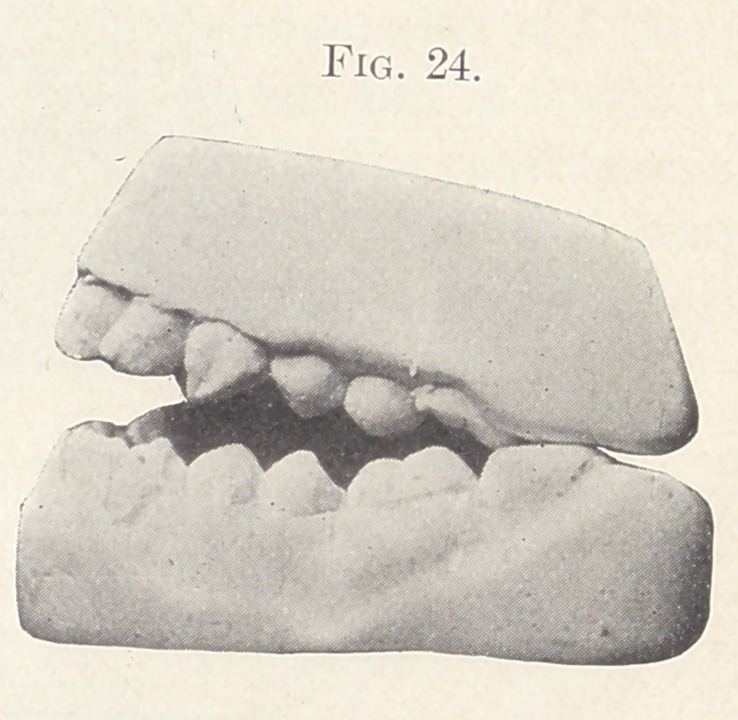


**Fig. 25. f25:**
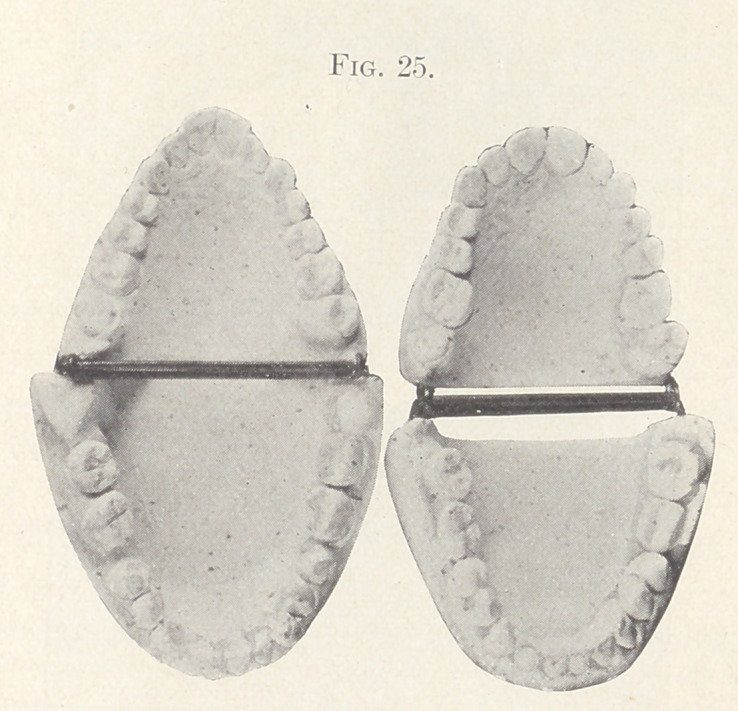


**Fig. 26. f26:**
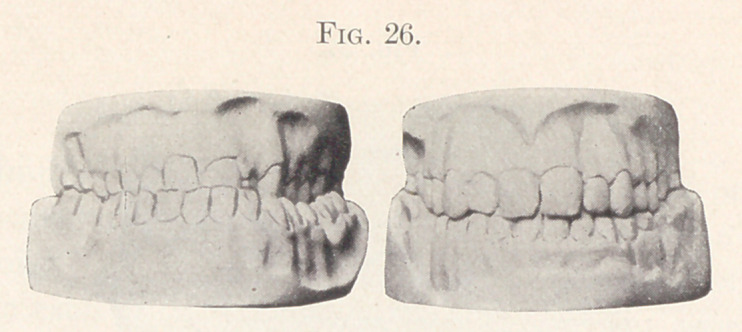


**Fig. 27. f27:**
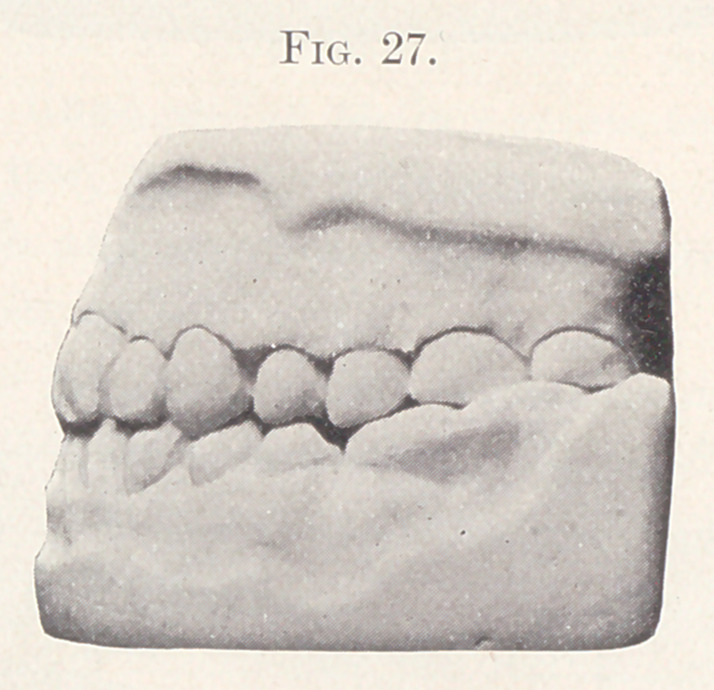


**Fig. 28. f28:**